# Ethnomethodological conversation analysis and the study of assemblages

**DOI:** 10.3389/fsoc.2023.1206512

**Published:** 2023-09-25

**Authors:** Pirkko Raudaskoski

**Affiliations:** Exploring Methods for Participation and Dialogue in Communication Research, Department of Communication and Psychology, Aalborg University, Aalborg, Denmark

**Keywords:** EMCA, heterogeneous assemblages, method, sociomaterialism, complexity, complicatedness

## Abstract

The material turn has challenged traditional social scientific and humanistic research approaches. Both individual and community are rejected as a starting point for theorizing what is going on in societies and cultures. In fact, all dichotomies are deemed suspect, and the research focus draws heavily on actual practices. The concept *heterogeneous assemblage* is used in at least two strands of the material turn with slightly different takes on the entangled nature of practices. These are actor-network theory, ANT (*cf.* STS, e.g., Callon, Latour, Law) and new materialism(s) (*cf.* process philosophy, e.g., Deleuze, Guattari). Both can be placed under the umbrella term sociomaterialism. In their analysis of concrete phenomena, Deleuzian assemblages tend to focus on embodied sensations (affect) that have rhizomatic threads of connection, whereas ANT’s assemblages include how heterogeneous entities (actants) stabilize certain practices. With a revised understanding of how the world works (ontology), the usefulness of traditional research methods (epistemology) to study concrete phenomena has also been questioned. Margaret Wetherell has suggested that affect assemblages can be analyzed as observable social practices, giving an EMCA-based study as an illustrative example. The question is whether both new materialist intensities (*cf.* certain approaches in psychology) and ANT’s connections to other people, places, and practices (e.g., in organization studies) could be analyzed with an EMCA approach. This paper acknowledges the existing possibilities EMCA offers to analyze heterogeneous assemblages as situated interactional and material entanglements and enlarges the repertoire by focusing on 1) how the material specifics can make the EMCA “why that now” analysis connect to larger assemblages than the local accomplishment of action, and 2) how observable orientations to phenomena outside of the situation can be treated as an assemblic activity. It will do this with 1) Goodwin’s concept *lamination* that enlarges the strictly situation-bound contextual configuration analysis to the cultural-historical formations through the use of material tools, and with 2) mentionings that combine Membership Categorization Analysis and Cooren’s interest in non-human (material) actors. In other words, the well-known sociomaterial concept *material-discursive* is translated into two analytical possibilities to study sociomaterial heterogeneous assemblages. An empirical study illustrates the tools in practice.

## 1. Introduction

Oxford Dictionary of English (Stevenson, 2015) defines the word “assemblage” as follows:

assemblage.

▶ *noun* a collection or gathering of things or people: *a loose assemblage of diverse groups.*

- a machine or object made of pieces fitted together: *some vast assemblage of gears and cogs*.
- a work of art made by grouping together found or unrelated objects.
- [mass noun] the action of gathering or fitting things together: the assemblage of electronic image and text databases.

While dictionaries concentrate on the original meaning of collection or gathering, researchers contributing to Wikipedia’s definition of the various forms of assemblic (an adjective not in the Oxford English Dictionary) thinking state: “Its central thesis is that people do not act exclusively by themselves, and instead human action requires complex socio-material interdependencies.” In other words, assemblic thinking concerns heterogeneous assemblages. The concept regards influences of various origins and types that are at play in any given situation or phenomenon, and the effects that emanate from it. The learning researcher Fenwick chooses the concept *sociomaterial* to cover various strands of assemblic thinking that “focus on materials as dynamic, and enmeshed with human activity in everyday practices” (Fenwick, 2015, p. 85). Assemblage is a concept used both in Actor-Network Theory (Latour, 2005) and the process philosophical new materialism(s) with a strong Deleuzian influence. Both focus on the actual going-ons in the world and how non-present forces play a role in them.

Everyday practices are at the very heart of studies in Ethnomethodological Conversation Analysis (EMCA), too. Garfinkel’s ethnomethodology insists that we stay close to the actual practices instead of considering them as realizations of abstract theoretical concepts. In fact, Garfinkel also approached the widely appreciated social scientific methods as studiable practices. In a similar though more abstract fashion, Fox and Alldred (2017) go through from a sociomaterialist (new materialism) perspective the typical social scientific methods (“research assemblages”) regarding everyday practices (“event assemblages”), to show “what research actually does” when the two assemblages entangle (p. 175). The vigilant researcher can then combine the existing methods as they deem best. Fox and Alldred see a tendency among sociomaterialist researchers to use qualitative methods, which is understandable considering that sociomaterialist theory focuses on the embodiment of participants, the different modes of language use, or what a material setting affords or connects to. However, EMCA is not listed as one of the qualitative methods; only Garfinkel’s (1984) “experiments with trust” is mentioned to find out how small changes can affect order production. There is certainly an analytic gap to be filled, especially because traditional sociological studies lack close analysis of the effect of materiality in ongoing practices.

Whether the existing methods are enough to answer the theoretical focus of sociomaterialist studies can also be approached from the perspective of how sociomaterialism (new materialism, posthumanism) has disturbed (inter- and trans) disciplinary thinking. For instance, Pennycook (2018) considers applied linguistics as an *epistemic assemblage* that gains from broader epistemic shifts in research interests rather than disciplinary categories. For Pennycook, sociomaterialism as the latest episteme means a totally new way of understanding and researching language use: “By stepping out of the humanist constructs of culture and nature, the individual and the social, and looking instead at the notion of distributed language and spatial repertoires, we can come to a new understanding of the materiality of language and social action” (p. 121). In environmental education research, Gough (2016) regards *postparadigmatic materialisms* as a necessary next step if the material place and its objects are the focus of empirical research. Both examples concern what two feminist science and technology studies scholars, Barad and Haraway, call diffraction: how phenomena arise and what they impact goes across disciplinary boundaries.

Charles Goodwin, a member of the EMCA community, could certainly be categorized as a researcher with a postparadigmatic and postdisciplinary take. In Goodwin’s last major publication, Co-operative Action (2018), the impact from various disciplines on his anthropological background becomes clear. He has, among others, several references to Latour and Ingold, the latter a fellow anthropologist for whom Deleuze’s process philosophy has been an important source. From early on in his career Goodwin challenged the strict division into linguistic, material, and visual anthropologies (e.g., Goodwin, 2000) which explains his awareness of various sociomaterialist researchers (e.g., Goodwin, 1994, 1995), even if he seldom referred to them as major influencers of his thinking (e.g., Goodwin and Salomon, 2019). However, his studies of how the material environment forms interactions (e.g., Goodwin, 2002) could be regarded as assemblage analysis. That is, in sociomaterial terms, they show how material things are performative (Fenwick, 2015). Goodwin’s analytical orientation to the material world, along with the sociomaterialist theorizing, has been a big inspiration for my own research (e.g., Raudaskoski, 2010, 2020, 2021a).

Goodwin founded a multimodal version of EMCA as a robust method to analyze what takes place in what Fox and Alldred call event assemblages. EMCA has also had its epistemic shifts to study how things get done in practice from language-based (with all the semantic and prosodic nuances) production of social order to how embodiment and other materialities shape that order. EMCA has recently become interested in touch (Cekaite and Mondada, 2020) and taste (Mondada, 2021) as publicly observable parts of the complex event assemblage-in-progress. The broadening of analytical interests has without a doubt coincided with the development of the data collection technologies as part of the research assemblage (see Erickson, 2004 for a historical account; McIlvenny and Davidsen, 2017 for a big video manifesto, and Raudaskoski, 2024, for what team camera work means for the transparency of data (collection) in empirical study). In the 1990s, Charles and Marjorie H. Goodwin were part of the Xerox Parc workplace studies where complex airport control room work practices with technological artifacts were studied closely. In other words, they studied how the materials were “enmeshed with human activity in everyday practices.” Lucy Suchman, an anthropologist and science and technology studies (STS) scholar, was the leader of the project. She has from early on combined the ethnomethodological approach with feminist sociomaterialist studies, which shows, for example, in references to the central feminist science studies scholars Barad and Haraway (e.g., Suchman, 2005). Another STS scholar with ethnomethodological background is Lynch (2015) who has become a regular contributor to practice theory publications that have a holistic and practice-based take on culture and society (*cf.* Reckwitz). In sum, sociomaterialist ideas have been incorporated in some EMCA research, but an equal awareness of the recent, multimodal versions of EMCA as method seems to be lacking in sociomaterialist studies.

## 2. Sociomateriality: the world and its research as assemblic entanglements

**Figure 1:**
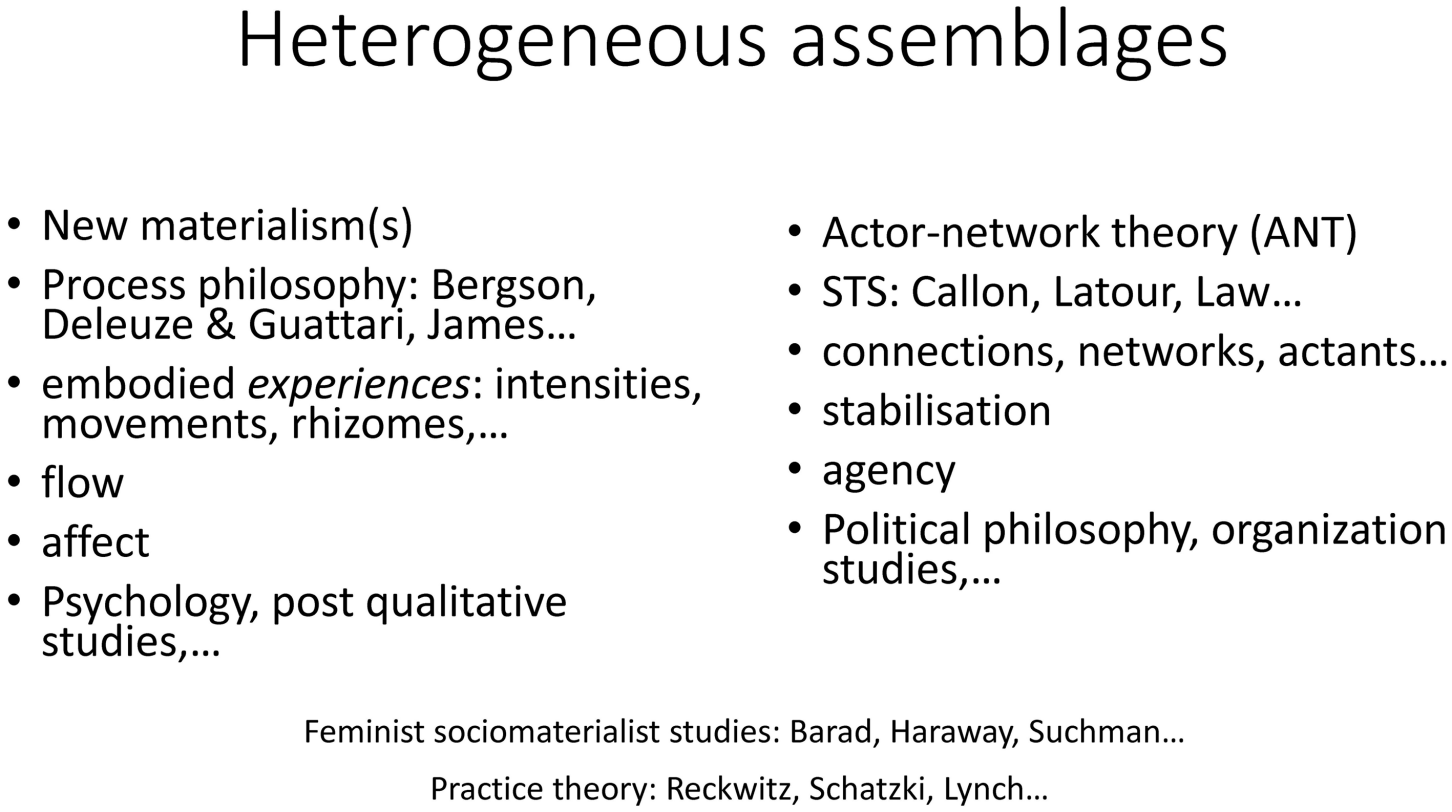
Two sociomaterial approaches to heterogeneous assemblages.

In the following, two major strands of sociomateriality, namely new materialism and actor-network theory, are given a short introduction. They are by no means monolithic approaches. Both shift the focus away from individual actors as the primary entity to study social scientific issues and both regard the material world as an agentive force. Therefore, these approaches are sometimes also labeled posthuman. Figure 1 depicts some of the core issues in the two approaches where heterogeneous assemblages are a central premise, both as regards to people and practices.

These approaches have different purposes, and neither has a strictly defined methodology. New materialism(s) grew out of the process philosophical thinking of, for instance, Bergson (1911), Deleuze and Guattari (1987), and James (1909/1996). New materialists focus on the often hard to explain situated embodied experiences that relate to ongoing phenomena in which different types of sensory experiences take place with connections to past experiences. Rhizome is used as a conceptual metaphor to highlight the possibility of having many types of varying linkages. The focus is on the ever-changing flow of lived life. Affect does not refer only to emotions, but to all sorts of intensities and movements. So, the left-hand side of the figure concerns individuals’ embodied experiences as assemblic entities: their life histories that invoke memories, their embodied sensations, their learnt ways of doing and saying in different activity types, and so on. In other words, the focus moves away from the individual as a separate entity to the individual as a heterogeneous assemblage of influences.

Actor-network theory (ANT) grew out of science and technology studies (STS) by researchers Callon (1991), Law (1991), and Latour (2005). They highlighted the ultimate interconnectedness of any phenomenon with its network of actants (people, objects, places, ways of doing things, etc.) to other practices, places, and people that have affect each other, that is heterogeneous assemblages. The role of nonhuman things became pivotal as they often are results of translating human practices to actants that also (like humans) make various types of actions and practices possible (or not). The main interest was to detect the development of stable positions in a network; how doing things in a certain way are treated as normal or even inevitable. Agency refers to the interest in the effects that actants have in different constellations. Also, Latour’s interobjectivity (Latour, 1996) considers the material environment as “timeshifting” to other places, practices, and participants through the history of making objects and placing them in the setting. Again, interobjectivity does not highlight just humans, but all forces that influence a situation.

Next, I go through Karen Barad’s (they, them, their) approach to materiality in more detail, as they have shown how the very basic ideas we have about materiality, gained through scientific evidence, depend on material arrangements. This is why I referred to them as a central theorist in my assemblic analysis (Raudaskoski, 2021b) of an experimental interdisciplinary workshop about the concept of abduction arranged in a Viking museum. Their *agential realism* treats matter as one of the aspects of the world that is in continuous becoming through various “practices of knowing”: “knowing is a matter of part of the world making itself intelligible to another part” (Barad, 2007, p. 185). Agential realism originates from Barad’s background in quantum physics and especially from Niels Bohr’s insights about nuclear science: The results about the material world depend on the material apparatuses that are used to measure phenomena. Therefore, instead of just measuring, these scientific apparatuses produce certain material realities (e.g., whether light is composed of particles or waves depends on the material measuring setup). Barad expanded the contextual impact to the larger institutional and political setting, showing the complexity of what influences the outcomes and impacts of any type of science. Situated emergencies link through entanglements to other complex circumstances. It is easy to see a connection to assemblic thinking: Agential cuts concern both local and larger assemblages. Agential realism connects phenomena in laboratories or other (research) sites to a myriad of entanglements (*cf.* Latour, 1983). It is understandable why Barad is also popular among practice theorists who write about *practice bundles* (*cf.* Schatzki, 2019), how practices connect to other practices, and how flat ontology therefore works. In other words, ‘micro’ and ‘macro’ are seen as useless vertical categories, in the same way as for Latour, who considered horizontal associations stabilizing as practices that connect to each other. For Deleuzian process philosophy, the flat ontology is locally constituted which shows in the rhizome metaphor (in contrast to a vertical tree). Hence, Barad’s materialist approach is among those that theorize how ordinary practices are constituted and the way they are involved in constituting larger issues.

One of the aspects that has been claimed as a crucial difference between the two sociomaterial approaches (Figure 1) is whether assembling focuses on the actual (longitudinal) processes of how things connect (new materialism) or on the nodes in the network: what is connected, and which translations have taken place (ANT)? Therefore, any concrete situation can be understood as a result of various types of emergencies (during the event or longer histories of the relevant entities) or a collection of nodes that relate to each other. In the following section, I discuss, among others, how these differences relate to EMCA studies and Wetherell’s solution to Deleuzian assemblage analysis.

## 3. EMCA for assemblage analysis

According to Barad, how evidence is achieved in empirical research is not just an analytical question, but a theoretical one as well. Social scientists and humanistic scholars are highly aware that research results depend on the chosen methodology. It is possible that the EMCA scholars have not considered the material tools, even though the measuring devices – that is, data collection technologies (from audio to video recordings) – have clearly contributed to our understanding of how “conversation” works as a materially situated, embodied phenomenon. Barad’s agential realism and an EMCA approach come close in their claim that phenomena (for ethnomethodology it is social order) are in the making all the time, and that we produce a variety of entities through material-discursive agential cuts where certain things are included while others are excluded. Barad emphasizes this by calling what is going on as intra-actions instead of interactions (which assume predefined entities). The fundamental idea in CA and ethnomethodology is similar: Practices constitute situations, identities, and so on. Furthermore, the ethnomethodological principle of approximation, that none of these have predefined, fixed, meanings, fits well with Barad’s theoretical concept of indeterminacy (vs. uncertainty) that gets resolved temporarily in practical action (*cf.* sequential turn-taking in EMCA). In my analysis of a phone call about a child-in-referral in a documentary on transnational adoption (Raudaskoski, 2010), I explored the methodological possibility to analyze Baradian intra-actions with multimodal EMCA as method. The analysis depicted how an identity translation of the future family members gets constituted through the use of embodied, material communicative resources and affect displays. The event involved various types of material-discursive inclusions and exclusions that also related to past private (e.g., through memory work) and institutional (e.g., through the official documents about the baby) circumstances. The paper also discusses the status of documentary as data that is a result of media professionals’ work practices, where their cut of the phone call was a result of a complex entanglement of both media production and societal concerns.

EMCA has shown its strength as a tool for empirically analyzing social practices as co-operative accomplishments from the perspective of communicative resources of participants. When talk-in-interaction is researched, participants’ past histories are indirectly present, though an EMCA analysis only deals with publicly available orientations to them. The growing awareness of the importance of embodiment and other types of materiality (of language, body, and the material surroundings; *cf.* Charles Goodwin’s contextual configuration) has resulted in “conversation” being replaced by “multimodal interaction” in certain versions of EMCA. Embodied participation reveals some of the learned ways of attending a situation, and the material setting on its part connects to past practices. Participants have changed from talk-based interactional partners to embodied (material) beings. Therefore, reflexivity does not only concern turn-by-turn production of meaning and, with that, intersubjectivity, but embodied other(s) and objects also participate in the reflective constitution of what is going on. In that way, Barad’s coinciding of relational ontology and epistemological processes, onto-epistemology, can be studied with multimodal interaction analysis: “Practices of knowing and being are not isolable; they are mutually implicated” (Barad, 2007, p. 185).

EMCA started with the analysis of talk as a way to achieve intersubjectivity and to get things done. With multimodal interaction analysis, not only embodiment but also Latour’s interobjectivity is as important. For example, we should ask what emerges out of the encounters with the nonhuman, sometimes language using, objects (*cf.* Raudaskoski, 2003)? Intersubjectivity in EMCA works through indexicality, the ongoing sense-making exercise that human members participate in in event assemblages. The ethnomethodologist Goode (1994, p. 102) expands the notion of membership to be that of the wider world and with that the notion of intersubjectivity. For Goode (2006, p. 90), intersubjectivity is not just based on language or culturally accepted behavior, but on sensual intersubjectivity, which includes all forms of living creatures. His approach comes closer to posthumanist theorizing. However, it is important to remember that sociomaterialism does not refute human agency, but asks us to take seriously, both in theory and in practice, how other materialities affect what is going on in the world. It could be claimed that the recent developments in multimodal interaction analysis provide a robust method to analyze event assemblages in their *in situ* heterogeneous becoming from the perspective of the forces (the affordances of humans included) that inhabit them.

In a multimodal EMCA analysis, collections and connections are in focus from the point of view of interactions. Linguistically oriented CA research is based on collections, that is, on how certain language forms function in talk-in-interaction and what their effects are in turn taking. Those results are valuable because they present a reliable analysis of what is going on moment-for-moment in the longer stretches of talk and other actions where the focus is on the effects of longer-term sequences, and how they affect or connect in a local sequence. Recently, there has been a growing interest in *longitudinal* CA studies (e.g., Pekarek Doehler et al., 2018; Deppermann and Pekarek Doehler, 2021) where the focus is on how the way of constituting the “same” interactional phenomena changes over time, rather than finding instances of similar shape. In their special issue Depperman and Pekarek Doehler give an overview of past longitudinal CA studies and divide them into development (child, learning), historical (ways of speaking over time) and joint interactional histories (families, organizations). The main focus is still on repeatedness, which leads them to study collections of how action formats change over time. The researcher’s work is to detect when a different looking realization of a phenomenon does the same work as a prior typical format of the phenomenon:

“It requires what Koschmann (2013, p. 1039) refers to as “same-but-different” analysis: To count as evidence for change over time, the phenomenon under scrutiny has to be different at time t2 from t1, yet similar enough to be interpretable as an occurrence of the same phenomenon—a token of the same type.” (Deppermann and Pekarek Doehler, 2021, p. 128).

However, assemblic analysis differs from such longitudinal studies because questions of (dis)similarity are not in focus. Instead, practices and participants’ experiences become central in trying to detect when (and not just how), from an assemblic perspective, actions connect. In other words, how does a phenomenon at time t1 relate to what is going on at t2. For instance, a prospective adoptive mother at t1 tearfully states that she is unhappy she cannot carry a child. At t2, when the couple hears over phone about the pregnancy of the biological mother of their future son, the husband glances at her and she silently cries. A statement produced in interview talk (t1) and embodied reactions during a phone call (t2) can be treated as connected, dealing with the couple’s life history about trying to have a child and (the husband’s awareness of) her pain for not experiencing pregnancy. This example deals with affect as assemblage. In Raudaskoski (2010) I referred to Deleuze and Guattari’s (1987) *mot d’ordre* as an explanation for the affective force of the word “pregnancy.”

**Figure 2:**
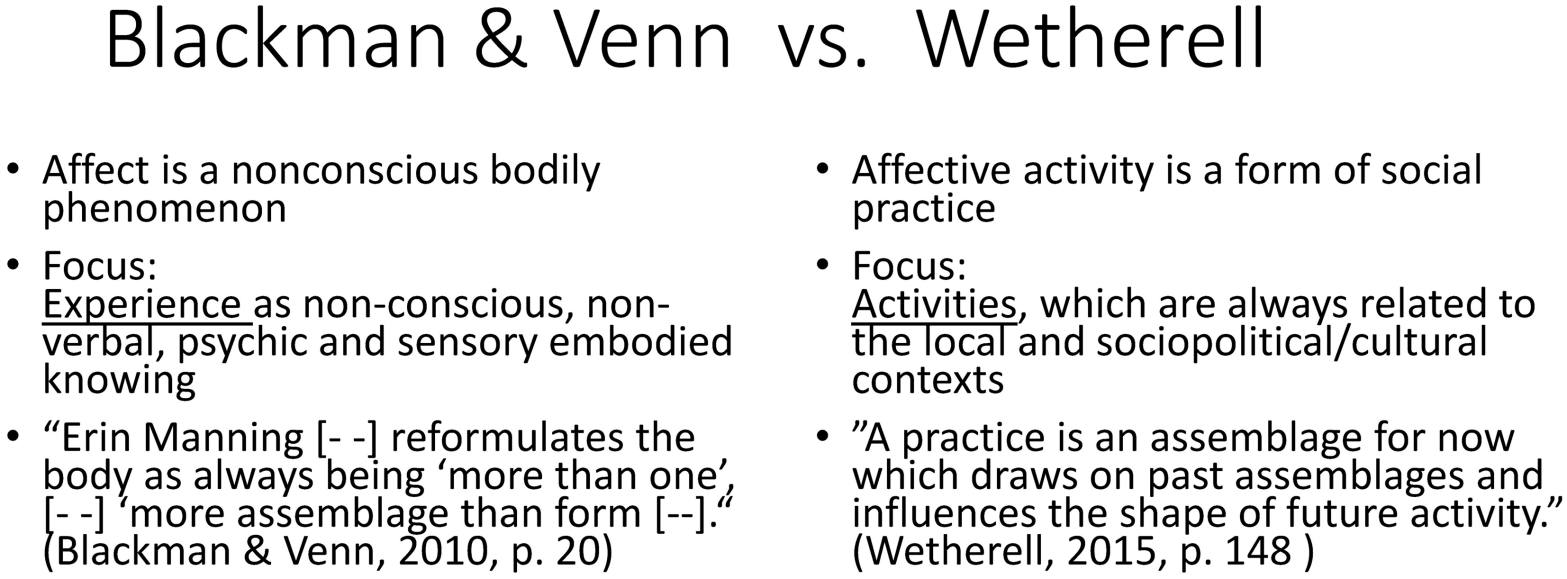
Two different takes on assemblages in psychology.

In psychology, Wetherell has criticized how the Deleuzian inspired approach to assemblages has been adopted in certain strands of psychology in relation to affect. Figure 2 describes the main differences between those, based on how Blackman and Venn (2010) and Wetherell (2015) approach affect. Wetherell’s interpretation of any social practice as a here-and-now assemblage that draws on past assemblages and has an impact on what happens next is similar to how multimodal EMCA analyzes actions in progress. In her earlier paper on the same topic, Wetherell (2013) used Marjorie Harness Goodwin’s analysis of girls playing hopscotch (Goodwin, 2006) as an example of how affect works as other-oriented social practice.

Charles Goodwin’s (2013) lamination depicts how the co-operative aspect of participation is always built on interactional or material substrate, adding thus to the local sense-making activity the aspect of sociocultural passing on of practices and the material tools involved in them. A material setting connects to the complex assemblage of knowledge, practical skills and actions needed to produce it, but also to occasions of participation in it and about it. This is why lamination, realized through contextual configuration, makes it possible to link to each other within one event the here-and-now continuously forming assemblages. The Goodwins and Wetherell were major inspirations in Raudaskoski and Klemmensen (2019) affect analysis of the participatory possibilities of a care home resident during an occupational therapy session: “With assemblage, the nature of affect as a complex relational phenomenon is accentuated, as it includes a multitude of effects of past assemblages. With emergence, the processual aspect of the ongoing situation as an assemblage drawing on past assemblages is foregrounded (*cf.* Wetherell, 2015)” (p. 161).

To sum up, multimodal interaction analysis, especially Goodwin’s contextual configuration, which orients to how participants use the material-semiotic resources in their action, is a robust analytical tool. It can be used to analyze Baradian intra-actions and affective assemblages because contextual configuration deals with the concreteness of attentive practice (participation frameworks). It can analyze what participants orient to moment-by-moment using language, body, and the material environment. However, the sociomaterialist approach connects the local sayings and doings in material environments to other places, people and practices, and regards these invisible participants as constitutive elements of any action, too. In Latour’s words:

“In most situations, actions will already be interfered with by heterogeneous entities that do not have the same local presence, do not come from the same time, are not visible at once, and do not press upon them with the same weight. The word “interaction” was not badly chosen; only the number and type of “actions” and the span of their “inter” relations has been vastly underestimated. Stretch any given inter-action and, sure enough, it becomes an actor-network.” (Latour, 2005, p. 202).

If “interaction” for Barad was not good enough because the concept assumes the interacting local entities beforehand, then for Latour it was not sufficient due to its narrow idea about what was impacting the ongoing situation. Above, I have tried to show how EMCA can be used to analyze intra-actions. In the following two sections I discuss two ways of dealing with larger entanglements.

### 3.1. Contextual configuration and lamination

With the larger assemblages, an important question is can we still do EMCA/multimodal interaction analysis or are we stretching the method to a breaking point? Goodwin’s assemblic lamination (how participants build on the other participants’ action – through contextual configurations) has a sharp focus on how participants co-operatively produce new knowledge through simultaneous and sequential action when they embodiedly laminate in concert with each other various means to constitute meaningful action with the help of the available material-discursive resources. He contends that:

“Complementary semiotic fields include 1) the mutual orientation of the participants’ bodies toward both each other, and the materials they are working with, which creates a public focus of attention and a locus for shared work; 2) language, including relevant deictic terms, organized within sequences of action within human interaction; 3) hands making environmentally coupled gestures (Goodwin, 2007a); 4) consequential phenomena in the surround that is being intensely scrutinized by the participants as part of the work they are doing together” (Goodwin, 2013, p. 16).

Lamination adds a historical aspect to local contextual configurations:

“Human beings build action by combining diverse resources (e.g., language structure, categories, prosody, postural configurations, the embodied displays of a hearer, tools, etc.) to perform both simultaneous and sequential transformative operations on a local, public semiotic substrate brought into existence by processes on many different time scales (from the immediately prior utterance to the progressive sedimentation of structure in tools, languages and settings)” (Goodwin, 2013, p. 41).

Thus, laminations are also larger assemblages in the sense that the semiotic substrate is not just local, but a result of various timescales. They are not directly visible in the local constitution of intelligibility and meaning, even if they can, in Latour’s words, be “interfered with by heterogeneous entities” (Latour, 2005, p. 202). Therefore, it is possible to claim that past places, practices, and participants are “present” when intelligibility is achieved (or not) in action in a co-operative setting. The most immediate and acceptable stretching of the EMCA/multimodal interaction analysis as assemblic method would be to (ethnographically) trace the participants. In an article about imagination, I traced relevant parts from a documentary that followed a couple in their adoption process (Raudaskoski, 2021a). Within a shorter timeframe (but with more complex data), I analyzed the doings of two participants during a nature hike, made possible by data from a camera team that used 360-degree cameras (Raudaskoski, 2023). As mentioned earlier, these types of longitudinal analysis would not focus on collections that would help understand how certain forms of language, gestures etc. are typically used, but how different types of participation support the analysis of encounters by the same participants, how they rhizomatically connect (*cf.* also Raudaskoski and Klemmensen, 2019). However, as I discuss below, the use of (especially human-produced) material things can be analyzed as connecting to the heterogeneous assemblages, even if the details of their production would not be available.

### 3.2. Mentionings: membership categorization analysis and Latourian organization studies

Another method many in the field of EMCA use is Membership Categorization Analysis, MCA (Jayyusi, 1984; Eglin and Hester, 1992; Silverman, 1998) which has been able to contribute to some aspects of the intelligibility, the situated concreteness and the ethnomethodological “why that now” of said or done as connected to large collectivities. For instance, what obligations and rights is a certain category expected to have. MCA starts with the implications of membership categories, where the analytical logic is different from EMCA’s focus on sequential interpretation and next turn proof procedure. However, identity analyses have been especially able to combine the sequential and turn-internal analyses to strengthen their points (*cf.* Stokoe, 2010). Thus, MCA adds to the analysis of the situated simultaneous embodied going-ons (*cf.* Goodwins’ research) the more general cultural and societal understandings of categories. I have used MCA to analyze, for instance, how a certain type of introduction to a white member sitting next to a transnational adoptee in a two-person jury in the final episode of a Danish version of the reality program Robinson from 2000 contributed to an amplification of attitudes:

“The growing methodological interest on how a real-life event can be linked to the cultural-historical spacetime (Agha 2007). It has been an attempt to dig into the possible formation of attitudes toward others outside of the realm of political (media) discussions” (Raudaskoski, 2011, p. 637).

Yet another way of doing assemblic analysis is to consider situated mentionings as participants’ orientations to other place/people/practices. This is what Francois Cooren, an organization theorist who combines Latour and Garfinkel in his analyses, has suggested: “interactions are never purely local, but dislocal, that is, they constantly mobilize figures (collectives, principles, values, emotions, etc.) that incarnate themselves in people’s discussions” (Cooren, 2010, description). Cooren regards Membership Categorization Analysis doing similar work, but he focuses more on the agency of the nonhuman assemblages than might be normally done in our MCA analyzes.

While Cooren combined EMCA and Latour’s actant analysis, Charles Goodwin had more subtle connections to Latour’s ideas. When analyzing the practical work of archeologists (e.g., Goodwin, 2010), he referred to Latour’s (1995) article on an interdisciplinary researcher group’s (him included) research field trip to Amazonas. Both Goodwin and Latour analyze how the scientists used the Munsell chart. On the other hand, as mentioned above, Goodwin appeared to be increasingly interested in the anthropologist Tim Ingold’s approach to anthropology (e.g., Goodwin, 2007b). Ingold has widened his references toward new materialist research (e.g., Ingold, 2013).

There are some theoretical interests shared between (multimodal) EMCA and the two approaches of heterogeneous assemblages. EMCA/multimodal interaction analysis can offer tools to study both local and larger heterogeneous assemblages and of both sociomaterial types depicted in Figure 1, a challenge that, for instance, Müller (2015) has discussed. In the following, I revisit the analytical practice with a data extract from a nature hike to showcase how to analyze both types of heterogeneous assemblages in an activity by combining Goodwin’s lamination (that enlarges the strictly situation-bound contextual configuration analysis to the cultural-historical formations through the use of material tools) with mentionings that combine MCA and Cooren’s interest in non-human (material) actors, and what can be inferred from these. The combination does not reveal all the possible assemblages that a situation has to outside of it (*cf.* Clarke’s (2005) mapping exercise to try and decipher heterogeneous connections), staying thus in the realm of human-centered agency and the intelligibility of indexical action that it is based on.

## 4. Danish nature days: why conservation?

**Figure 3:**
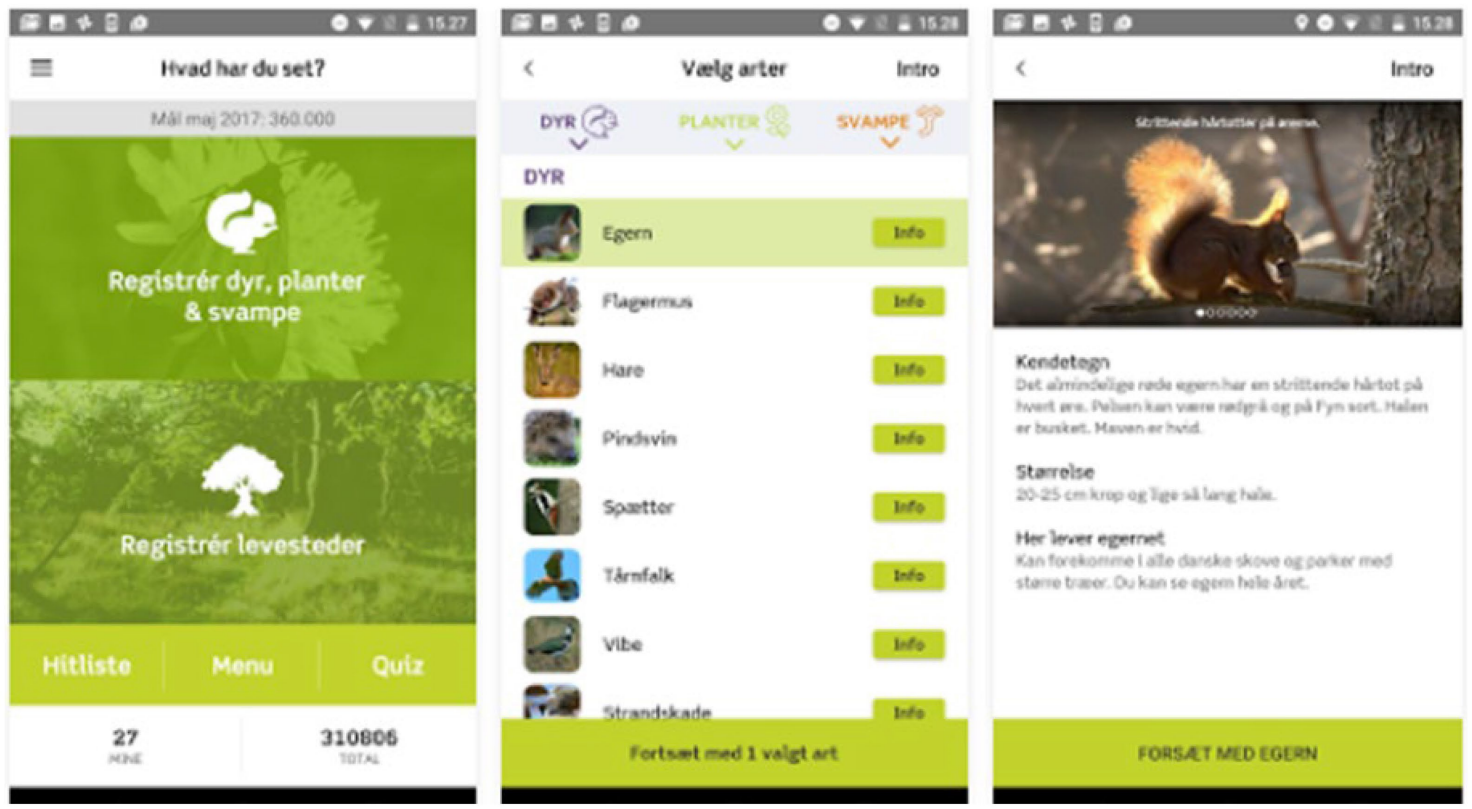
Three screens from the Danish “NatureCheck” app.

The data extract under scrutiny below comes from a hike called “Why Conservation” that was arranged during the very first (2016) Danish annual Nature Days public event. One aspect of the hike that got my analytical interest was why the participants seemed to be totally disinterested in an app that a guide introduced, even if they were in the nature where the app was meant to be used. The Danish Society for Nature Conservation (DSNC) had developed the app for nature goers to report on sightings. On the website of the (Google) app, they introduced the app as follows (see Figure 3): “NaturTjek [NatureCheck] is for you who wants to help study how the biological variation (biodiversity) is doing in Denmark, to learn about nature and to have fun while you are doing it.”

**Figure 4:**
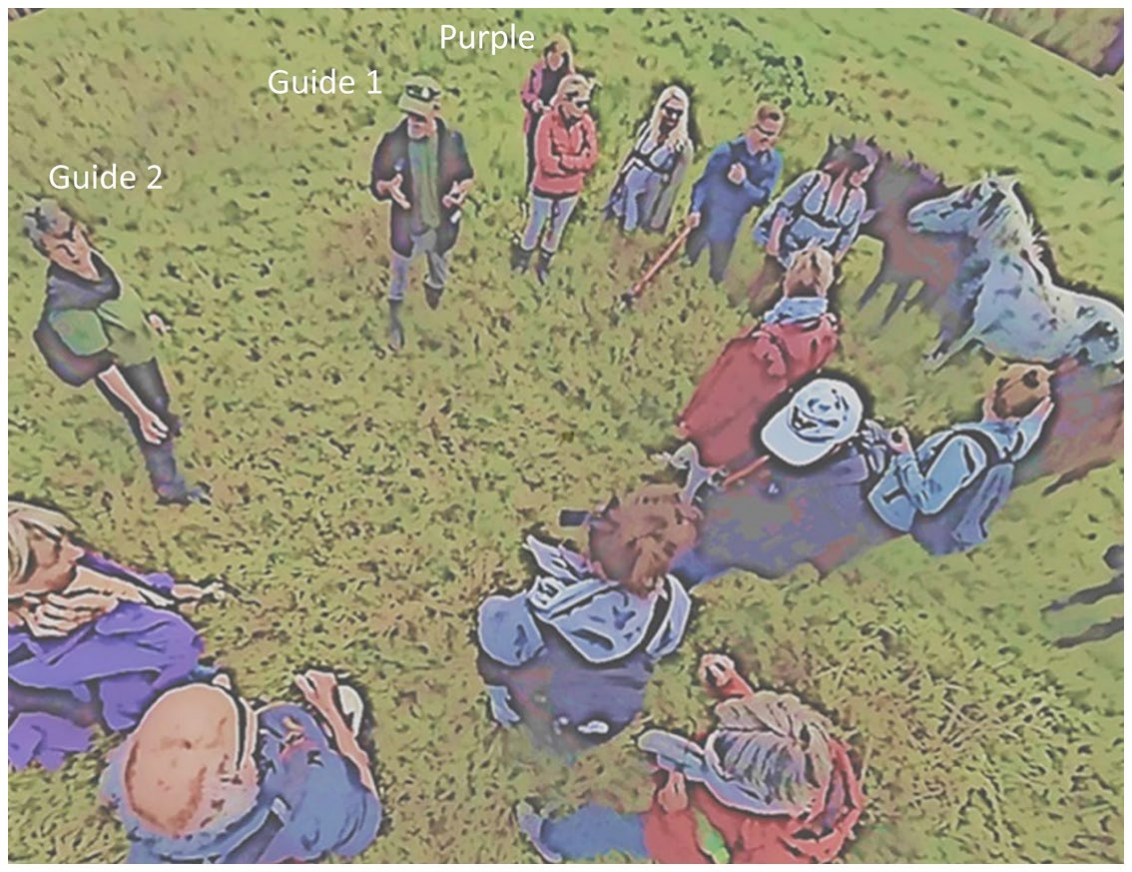
A group of nature hikers.

The app was part of a citizen science project run by Copenhagen University in cooperation with DSNC. By the time a guide introduced the app to the nature hike participants the group had acquainted with the swamp area they started the hike from, they had inspected some of the plants in that area and walked a bit further to the site where they were met by a pack of six horses. In the following, I examine the ethnomethodological *just thisness* that led to the introduction of the app to the group, how the group members react to that introduction, and what kinds of entanglements or assemblages may be discovered. In Figure 4 I have also marked who the guides are and a group member (Purple) who took up the topic in the first place (the hike was documented by a traditional 2D-camera, three chest-mounted GoPros, and two 360-degree cameras on poles out of which one made it possible to get a close-up near the ground (see the participant-researcher in blue) and the other from above the group, from which the shot in Figure 4 comes from).

**Figure 5:**
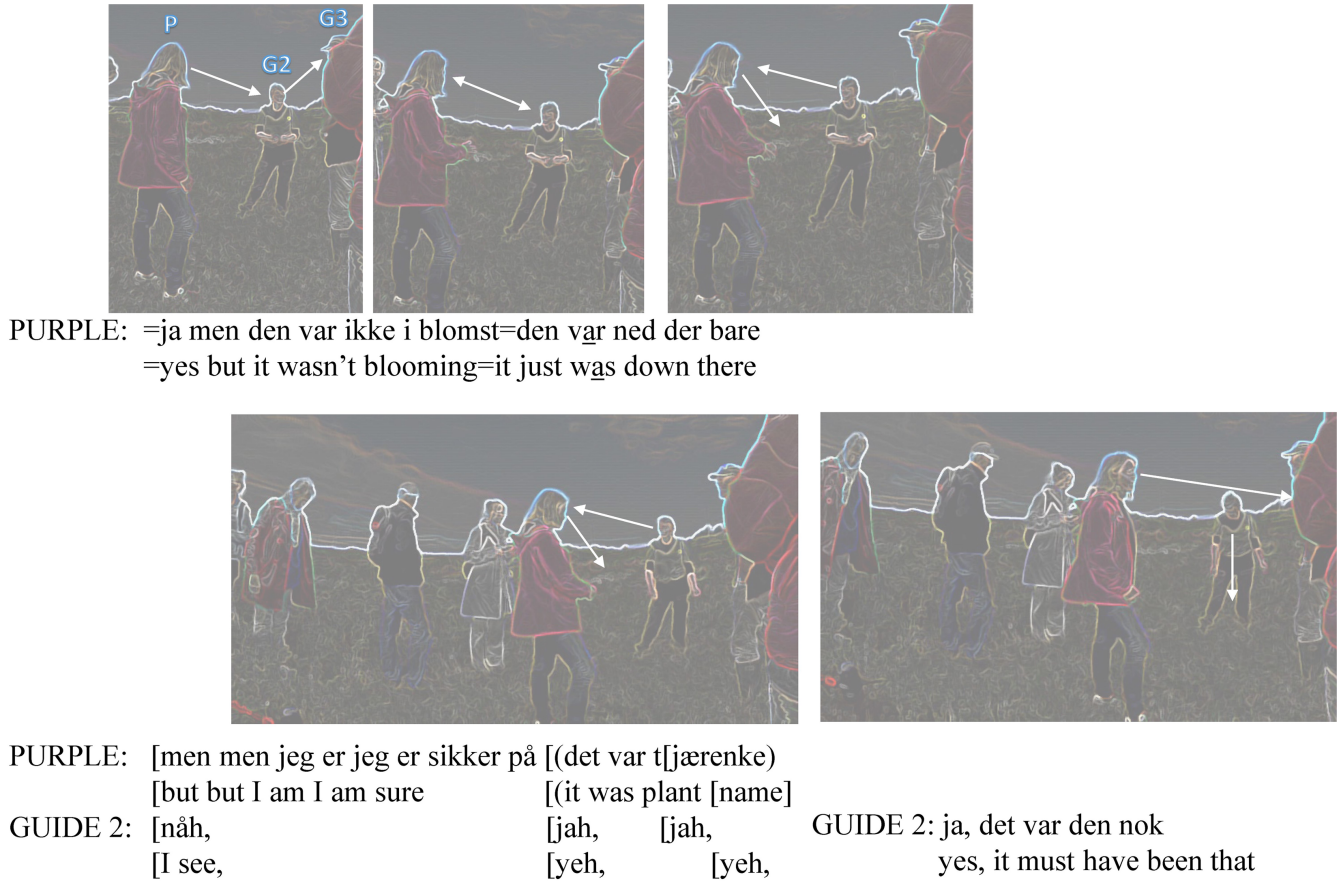
Reporting on a plant noticed earlier.

The transcription of how the introduction was set to proceed is a type of cartoon transcript (Laurier, 2014) with a Jeffersonian transcription of both the original Danish and the English translation. The stills show what is going on during the transcribed talk under them. We start with the group gathering to stop around the two guides. Purple has just arrived at the spot and reports having seen an interesting plant earlier (see Figure 5). Purple (P), Guide 2 (G2) and Guide 1 (G1) are marked again in the first pseudonymized frame. The white arrows depict the (sometimes mutual) gaze directions.

With Cooren we could call this mentioning of the plant *incarnation* (Cooren, 2010, p. 6), assembling the past observation of an object to the present situation. Purple is looking at Guide 2 while talking, constituting her as the primary recipient and, therefore, expert in the issue of the local plants. The other members of the group become overhearers. Purple must keep her distance from Guide 2 who is preparing to talk about the plants in front of her; she laminates her appreciative feedback to Purple with her primary situated task. When purple explains that the plant “just was down there,” her gaze shifts down, with her right hand in a loosely downward pointing fist. The incarnation becomes even stronger with the use of ‘Deixis am Phantasma’ (deixis in the imagination) (Stukenbrock, 2014). The last frame in Figure 5 shows Guide 2 having shifted her gaze from Purple to the plants in front of her (maybe because Purple still is orienting to the imaginary plant), and Purple’s head back from the body torque to look to the direction of her body posture.

**Figure 6:**
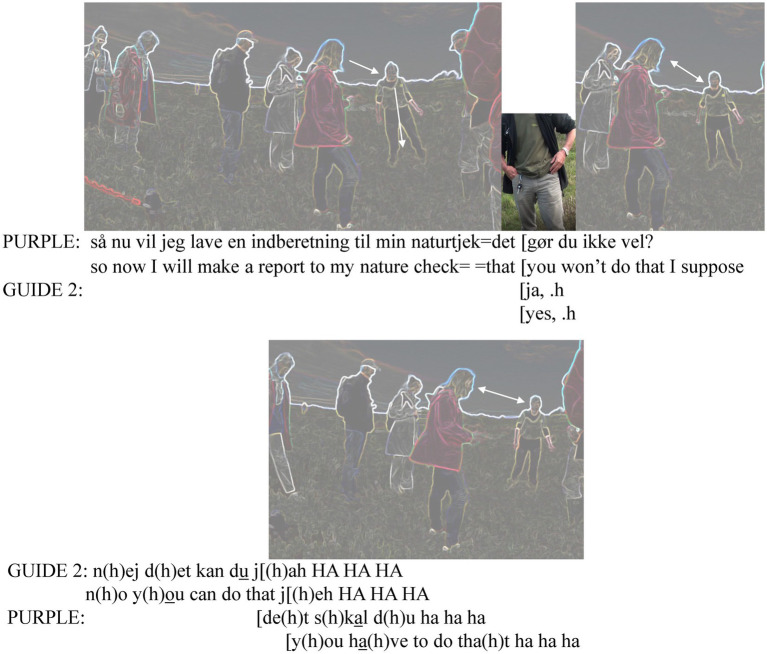
Topicalizing NatureCheck app.

After this (Figure 6), instead of walking ahead, Purple shifts her gaze back to Guide 2 and the focus of talk from the plant to registering the sighting to the NatureCheck app:

By calling it “my” app, Purple implies that she has been using the app regularly. Guide 2 acknowledges her expressed intention (“yes”), followed by a little inbreath that marks her readiness to start introducing the plants in front of her. However, Purple continues her turn, teasing the guide about her not doing it for her. This suggests she is familiar with the guide and her likely reluctance to use the app. Guide 2 agrees, shifting the responsibility to Purple (stress on “you”). Guide 2 does this with laughing tokens, concluding her turn with laughter particles. Purple joins her laughter while producing a mock request (“you have to”). In this brief exchange Purple has connected the app with the local plant, the latter of which is most probably of interest to Guide 2 as well. By engaging Guide 2 to the registration of the plant, Purple manages to link the app to the guide’s (lacking) user skills, a locally invoked assemblage again, but this time to the guide’s (in)competencies.

In MCA terminology, the teasing of Guide 2 could also be heard as invoking certain obligations for a nature guide: They should use this citizen science app and introduce it to others. Purple’s and Guide 2’s interaction intertwine epistemic and deontic authority (*cf.* Stevanovic and Peräkylä, 2012) from shared knowledge of the plant to a humorous exchange where both parties express the right to decide who should register the sighting. Meanwhile, Guide 2 has been waiting for a chance to start talking about the plants in front of her, so her situated responsibility is connected to that, instead. That ongoing obligation could be part of the reason for Purple’s statement (“you will not do that I suppose”) which gets a laughter-filled answer from Guide 2 (“no you can do that”), the line (Goffman, 1967) of which Purple continues in her mock direct order laminating it through format tying to Guide 2’s turn (“you have to do that”). Even if it can be heard as teasing or general verbal play, it still is a reminder of the duty that a nature guide should have: doing being an example of a good nature protector.

**Figure 7:**
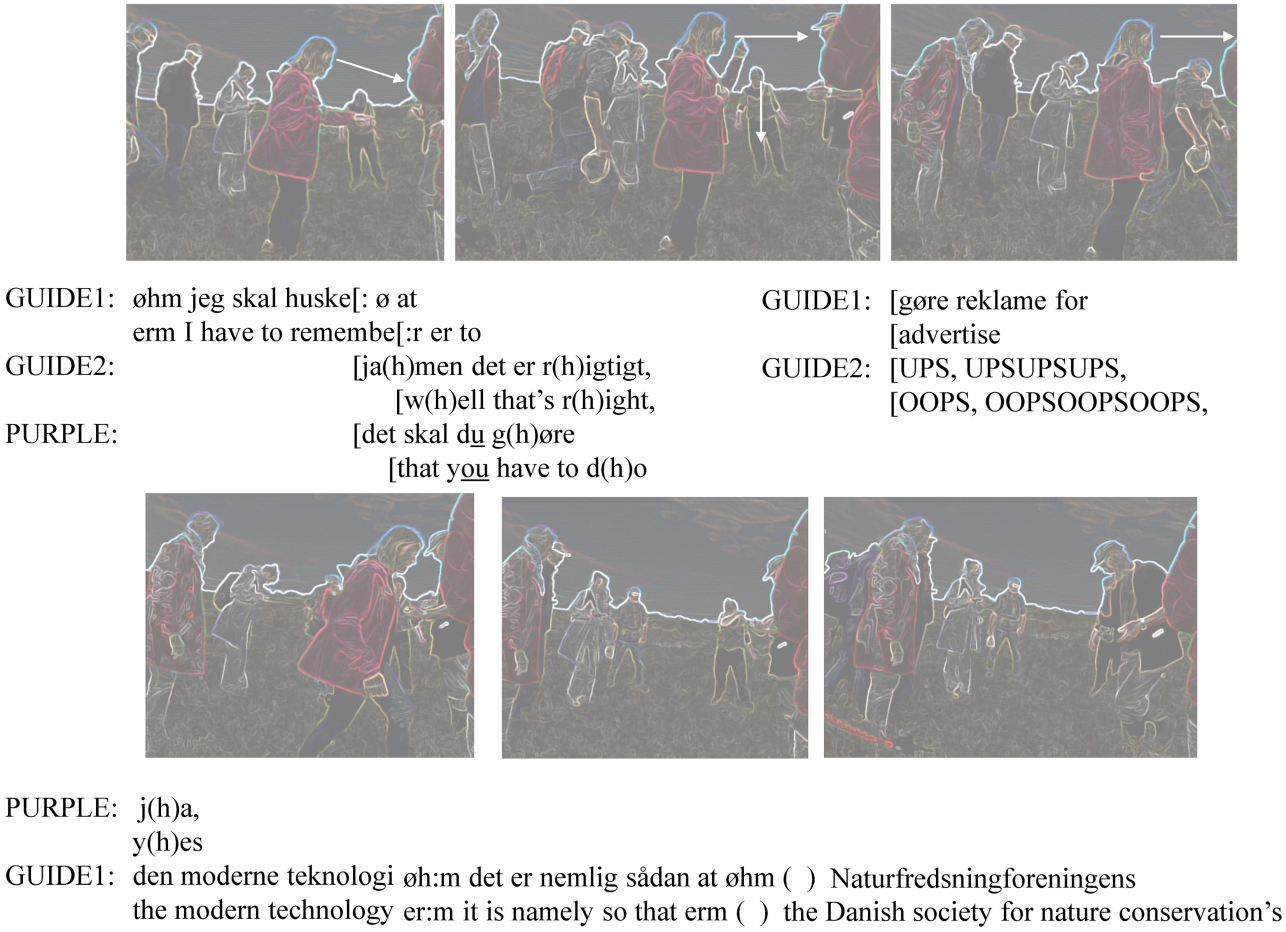
Start of longer app introduction.

By the time the short dialogue about the plant and its registration to app was over, Purple had turned her gaze to the male guide in front of her. Already Purple’s first mention of the app (Figure 6) had occasioned Guide 1 to take out his smartphone from the left front trouser pocket. He starts the introduction to the app after Purple turns to face him (Figure 7).

Guide 1 starts his introductory talk to the app by verbalizing the obliged nature (“I have to remember”) of the “advertising.” This incarnation of a previous plan or agreement is different from how he would start talking about the features of the immediate setting, that is, reoriented to a specific feature of it (*cf.* De Stefani and Mondada, 2014). In overlap with Guide 1’s lengthened “remember” Guide 2, still laughing while talking and gazing down at the plants in front of her, acknowledges Purple’s demand for her to use the app. By then Purple has already turned to Guide 1 who has the phone in his hand. Purple now addresses him with the same tone and with a stress on “you.” In other words, Purple now turns to him as a nature protection hike guide with the membership obligation to register biodiversity. This is at a large assemblic scale, whereas Guide 2 is doing a very local type of nature protection: She stops people from stepping on the rare plants in front of her (“OOPS, OOPSOOPSOOPS”).

**Figure 8:**
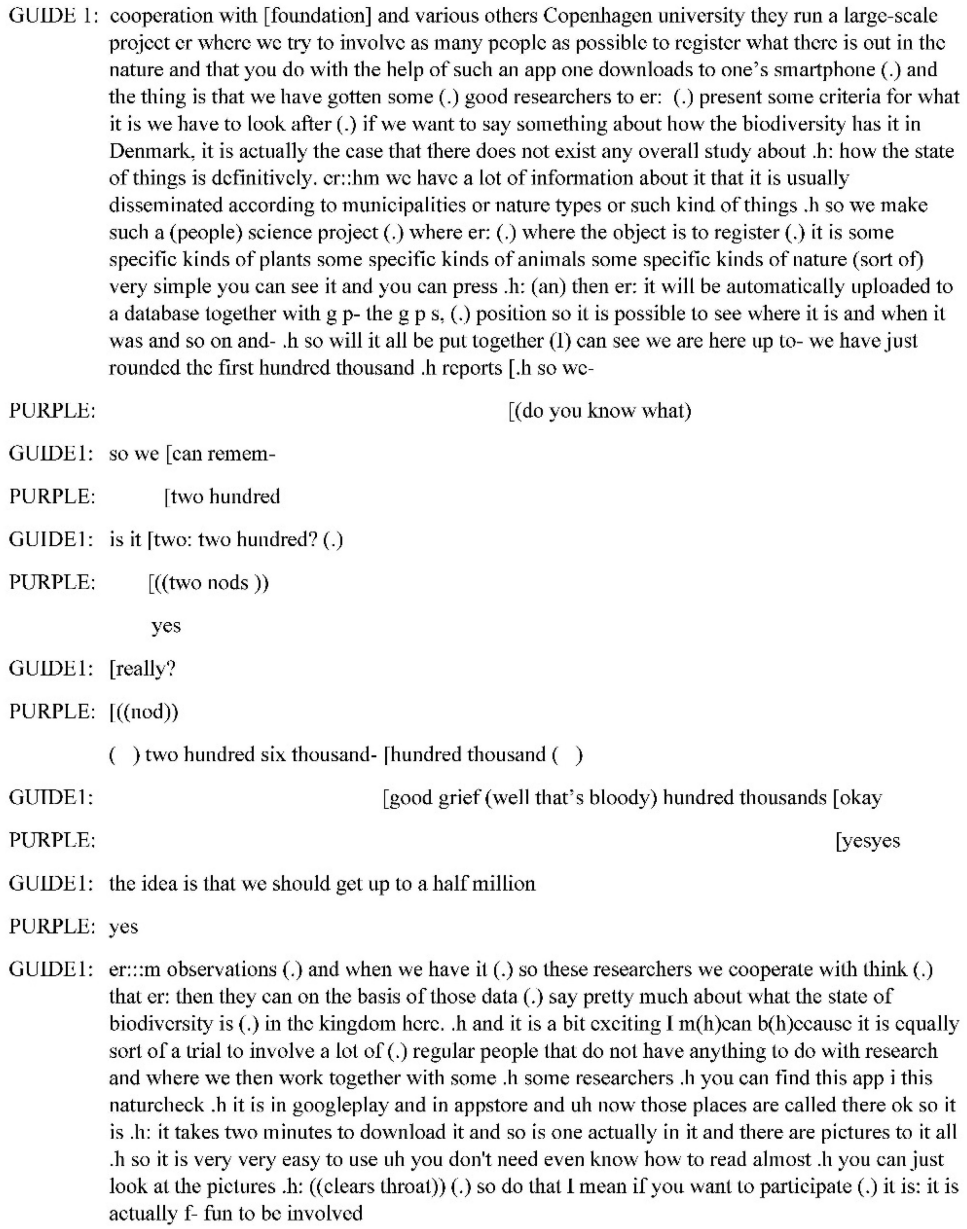
Guide 1 introducing the app.

Figure 8 shows the English translation of the rest of Guide 2’s long introduction to the app. The guide starts from the history of how the app came about and the purpose of it, which is to register the state of biodiversity in Denmark.

The Guide names the same NGO that has arranged the hike as the instigator of the project, thereby laminating the purpose of the app to the situation at hand. The “good researchers” are clearly concrete people with names that Guide 1 as one of the “we” knows, but they are incarnated to the description as an anonymous group. While the guide is speaking, the participants exhibit little visible interest in the app introduction (*cf.* Figure 4 which shows the moment when the guide says “overall study”). Purple is the only one who is actively involved by correcting the guide about the number of sightings she can see from the app on the smartphone. The other participants gaze around, pet and chuckle at the horses who come to them for attention, chat about them, and so on. The practical explanation might be that the participants are unable to see what the guide sees on the screen, a condition that Raudaskoski et al. (2019) call bystander ignorance; they are not invited to look at the app in Guide 1’s hand. They might also see the app presentation as an interruption to Guide 2’s already started orientation to the plants in front of her. Towards the end of his presentation, Guide 1 glances twice at Purple, indicating that she is the “reason for” the advertisement.

Guide 1 finishes up (not in transcript) by turning to Guide 2 and repeating how he started the introduction: It was good to get the advert of the day done, and his colleague agrees. By calling his presentation an advert again, the guide constitutes it as something he ‘had to’ remember to do. The uninterested audience might be a reason for packaging the presentation as an aside, even if he tried to make the actual use of the app sound more interesting (“fun”). Guide 2 commences talking about the plants in front of her, showing her main focus in the situation. Purple then uses a horse approaching her (phone) to connect to the guide’s “advert” in a humorous way, claiming that the horse wants to register as an app user.

## 5. Discussion of the app introduction

The assemblage for now is the local accomplishment of action through talk, embodiment, and use of the material environment and that achievement draws on past assemblages: the participants’ past experiences, the type of situation they are in and the material setting with its discourses. So, when Guide 1 turns, thanks to Purple’s occasioned reminder (via Guide 2), to the app on his phone, he turns to the distant DSNC and Copenhagen University and to the local Danish nature around them, together with the nature lovers that have come to the nature hike, and the team of video researchers who were recording the event.

Thus, when the two “infrastructures” – that of a nature hike and a scientific citizen project about the same plants, animals and sites – meet, there are only two participants (Guide 1 and Purple) that have opened the app, connecting to the many assemblages with that action. The aim of the app is to produce epistemic representations, “big data” (tokens of types), it is working for a center of calculation (Latour, 1987). Its goal is to engage citizens in biodiversity research through participation. Participants in a nature walk, on the other hand, have come to experience the immediate nature *via* their senses and to learn more about it personally; it is secondary to report on it; potential images are taken for private use. They also have two sorts of materiality as affordances for involvement in the situation: broad open nature to explore *via* embodiment and a little gadget with several stages to learn how to utilize. We could claim that Purple is trying to combine the experiences and their representations through connecting to the here-and-now noticings and interpretations of a (by now invisible) plant and an inquisitive animal in the environment. She is accomplishing the connection to the app through humor that makes use of the alternative focus that many of the participants oriented to while Guide 1 was talking, namely horses. We witness an attempt to enhance rationality (participation in citizen science) through affective activity.

A close analysis of how the app was introduced can thus tell us about the local effects of assemblages, how they affect our experiences and actions in our world. As De Stefani and Mondada remind us: “even stationary interactional spaces are dynamically assembled and constantly reassembled” (De Stefani and Mondada, 2014, p. 173). There can be complex assemblages that come into play in the situation. This makes the questions of epistemology and ontology blurred. How does getting to know nature while in it and a scientific citizen project about it come together or clash in the concrete situation? Maybe it has something to do with the different ontologies: while using the app could be categorized as a synthetic situation of use (Knorr-Cetina, 2009) with its dynamic figures, what makes encountering plants and animals in a nature hike persuasive is that they are ontologically stable entities. However, if the participants are interested in the topic of the hike, nature conservation (*cf.* ethico-onto-epistem-ology, Barad, 2007), should they then feel responsibility to register what they observe to a biodiversity project run by the same NGO? This question hints at flat ontology at work: interest in nature conservation is done by attending a hike arranged by the national society that has cooperated with a university to study the existing state of biodiversity, all of these aspects of the here-and-now and the larger issue of Danish nature conservation coming together in the app that is open in two smartphones. In this case, the local practice bundle did not connect so well, but it is only through somebody being in nature and reporting it through the app that the assemblage university-DSNC would be successful. Will non-attendance to the presentation protect them from feeling obliged to use the app for the rest of the hike and thus free them to enjoy the nature firsthand without having a smartphone in the hand to make a representation of the seen, heard, smelled, tasted, and felt? The guide is careful not to claim the right to request that the participants should download the app. He finishes his presentation after highlighting the ease of use of the app and the quick download time with a directive “so do that” but adding immediately the mitigating “I mean if you want to participate.” In other words, he does not claim any type of deontic authority over the participants.

The analysis presents an example of the intricacies of how an app is introduced (remembering to advertise), the materiality of the smart phone (hard to share/see), and the contextual configuration of the group (attending to rare plants in a circle; having a pack of horses to orient to) were some detectable reasons for why the nature around the participants won their interest over the nature app on a smartphone in the hand of a guide. However, the app was directly connected to the assumed interests of the group members: protecting the Danish nature. Therefore, the situation was also connected to a larger assemblage that entangled the participants in an ethico-onto-epistem-ological dilemma of participating citizens that did not want to become citizen science participants.

## 6. Conclusion: EMCA and assemblage analysis

The paper has explored the heterogeneous assemblage as a concept that is welcomed both in new materialist (psychological) research and in STS and organizational studies. EMCA/multimodal interaction analysis can give analytical tools for empirical studies from both perspectives, even though the first focuses on the inner experiences of participants and the latter on the describable relationships between components. In addition to the EMCA/multimodal interaction analyses, with touch and other sensory experiences included, that can benefit the study of complex situated assemblages, two possibilities for including the larger temporal or geographical sphere in the analysis of local phenomena were discussed in detail: 1) Goodwin’s lamination that enlarges the strictly situation-bound contextual configuration analysis to the cultural-historical formations through the use of material tools with 2) mentionings that combine MCA and Cooren’s interest in non-human (material) actors. In other words, the well-known sociomaterial concept of material-discursive is translated into two analytical possibilities. The analysis of a nature hike illustrates both: 1) How the app was, with all its connections and implications, laminated to the situation at hand; and 2) how a previously detected plant was incarnated by a hike participant to introduce the app as a topic. While the app was not of interest to the group, the reasons for the guide to introduce it and the way the lack of interest was exhibited could be connected to certain obligations as participants in a conservation nature hike.

The theoretical attraction, yet analytical difficulty, of assemblages is that they are heterogeneous, they cover diverse phenomena when analyzing situated practices from participants’ experiences and memory to institutional (family and others) histories to the material environment as complicatedness (Latour, 1996) that implies other places, participants, and practices. In their special issue introduction, Deppermann and Pekarek Doehler (2021) bring into focus the prior actions of social interaction that their different scenarios exemplify. Also, they all seem to point to socio-cultural/−historical approaches, something that for instance Charles Goodwin was very aware of. Latour (1996) contrasted complicatedness to complexity in order to highlight how the material aspect of the complexity of any ongoing event assemblage is connected to how those materials got to be in the situation in the first place. However, ANT has been accused of not concentrating on the practices that make the connections between the actants in the network. From an EMCA perspective, to better understand the connections, it would require longitudinal ethnographic studies where practices are followed closely (*cf.* sociocultural studies). If this is not possible, the multimodal interaction analysis toolbox could make use of the two suggestions for how to use lamination and MCA for assemblage analysis. Fenwick (2015) has criticized sociocultural participation approaches for their lack of taking the agentive role of materials seriously. Therefore, this paper hopes to add a link between EMCA and a multiplicity of sociomaterial and with that participant-oriented assemblage approaches.

## Data availability statement

The original contributions presented in the study are included in the article/supplementary material, further inquiries can be directed to the corresponding author.

## Ethics statement

Written informed consent was not obtained from the individual(s) for the publication of any potentially identifiable images or data included in this article because the images are pseudonymized. The participants were offered to undersign a written consent where pseudonymization was stated, but they refused the possibility and expressed clearly to the camera that they are happy with the collection of video data as long as it is used for research purposes only.

## Author contributions

The author confirms being the sole contributor of this work and has approved it for publication. Part of the analysis was based on a paper presented in International Pragmatics Conference, Belfast, 2017 by PR and Paul McIlvenny.

## Conflict of interest

The author declares that the research was conducted in the absence of any commercial or financial relationships that could be construed as a potential conflict of interest.

## Publisher’s note

All claims expressed in this article are solely those of the authors and do not necessarily represent those of their affiliated organizations, or those of the publisher, the editors and the reviewers. Any product that may be evaluated in this article, or claim that may be made by its manufacturer, is not guaranteed or endorsed by the publisher.
